# Insights into the toxicological effects of nanomaterials on atherosclerosis: mechanisms involved and influence factors

**DOI:** 10.1186/s12951-023-01899-y

**Published:** 2023-04-28

**Authors:** Siyu Chen, Yuan Su, Manjin Zhang, Yulin Zhang, Peiming Xiu, Wei Luo, Qiuxia Zhang, Xinlu Zhang, Hongbin Liang, Alex Pui-Wai Lee, Longquan Shao, Jiancheng Xiu

**Affiliations:** 1 Guangdong Provincial Key Laboratory of Cardiac Function and Microcirculation, Nanfang Hospital, Southern Medical University, Guangzhou, 510515 China; 2 Stomatology Center, Shunde Hospital, Southern Medical University (The First People’s Hospital of Shunde), Foshan, 528300 China; 3 Stomatological Hospital, School of Stomatology, Southern Medical University, Guangzhou, 510280 China; 4 Guangdong Medical University, Dongguan, 523808 China; 5 Prince of Wales Hospital, The Chinese University of Hong Kong, Hong Kong, China

**Keywords:** Nanomaterials, Nanotoxicology, Atherosclerosis, Endothelial cell, Smooth muscle cell, Immune cell

## Abstract

Atherosclerosis is one of the most common types of cardiovascular disease and is driven by lipid accumulation and chronic inflammation in the arteries, which leads to stenosis and thrombosis. Researchers have been working to design multifunctional nanomedicines with the ability to target, diagnose, and treat atherosclerosis, but recent studies have also identified that nanomaterials can cause atherosclerosis. Therefore, this review aims to outline the molecular mechanisms and physicochemical properties of nanomaterials that promote atherosclerosis. By analyzing the toxicological effects of nanomaterials on cells involved in the pathogenesis of atherosclerosis such as vascular endothelial cells, vascular smooth muscle cells and immune cells, we aim to provide new perspectives for the prevention and treatment of atherosclerosis, and raise awareness of nanotoxicology to advance the clinical translation and sustainable development of nanomaterials.

**Graphical Abstract:**

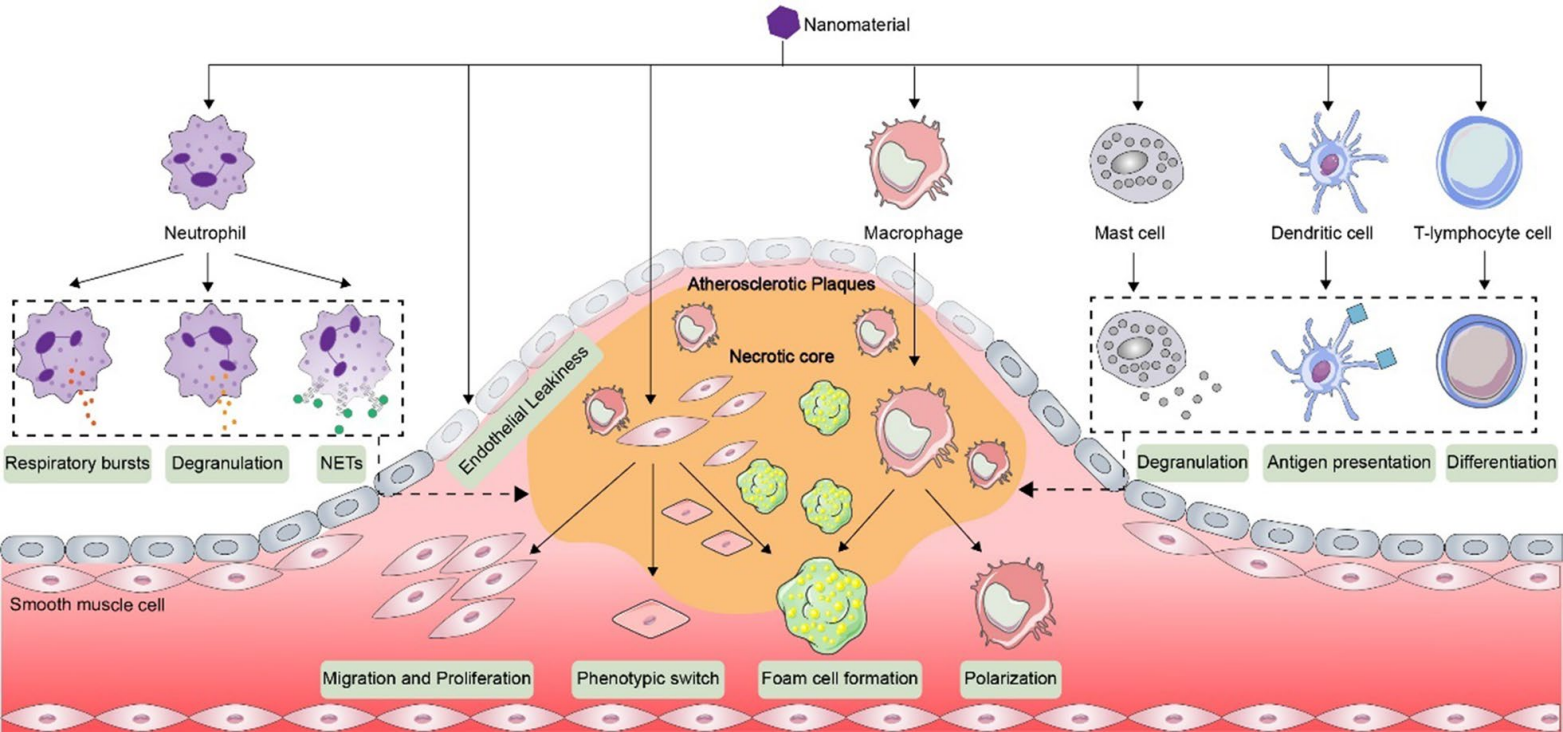

## Introduction

Cardiovascular diseases (CVDs) are the leading cause of death worldwide. In 2019, 17.9 million people died from CVDs, accounting for 32% of all deaths worldwide [1]. The most common and important pathological feature of cardiovascular disease is atherosclerosis (AS), a condition in which lipid accumulation and inflammation occur in large and medium-sized arteries, causing plaque formation. Atherosclerotic plaques occur in the subintimal space of blood vessels and are mainly composed of vascular endothelial cells, vascular smooth muscle cells, and immune cells such as macrophages. These cells cause plaque formation through lipid uptake, inflammation, and activation of immune system. Advanced plaques undergo rupture or erosion, leading to thrombosis, which blocks arteries and obstructs blood flow, eventually causing a series of life-threatening clinical manifestations, such as acute myocardial infarction, heart failure, and stroke [2]. Therefore, it is urgent to detect early abnormal and vulnerable plaques and to improve the clinical outcomes of AS. Nanomaterials (NMs) are materials at the nanoscale (1–100 nm). According to their chemical composition, NMs can be classified as polymeric, inorganic and lipid-based NMs. With the development of nanomedicine and nanotechnology, an increasing number of NMs have been applied in the delivery of antiatherogenic drugs, stent functionalization, vascular graft fabrication and imaging contrast agents [3]. For example, poly (lactic-co-glycolic acid) nanoparticles (PLGA NPs) lower low-density lipoprotein (LDL) and suppress the destabilization of plaques by delivering the clinical drug pitavastatin (PT) [4]. Magnesium fluoride nanofilm-coated stents reduce corrosion and improve stent efficacy [5]. A liposomal MRI Gd contrast agent is used for early atherosclerosis detection [6]. NMs are highly controllable compared to conventional diagnostic and therapeutic tools for AS and have advantages such as targeting specific tissues or cells by adjusting their physicochemical properties and surface modifications.

**Fig. 1:**
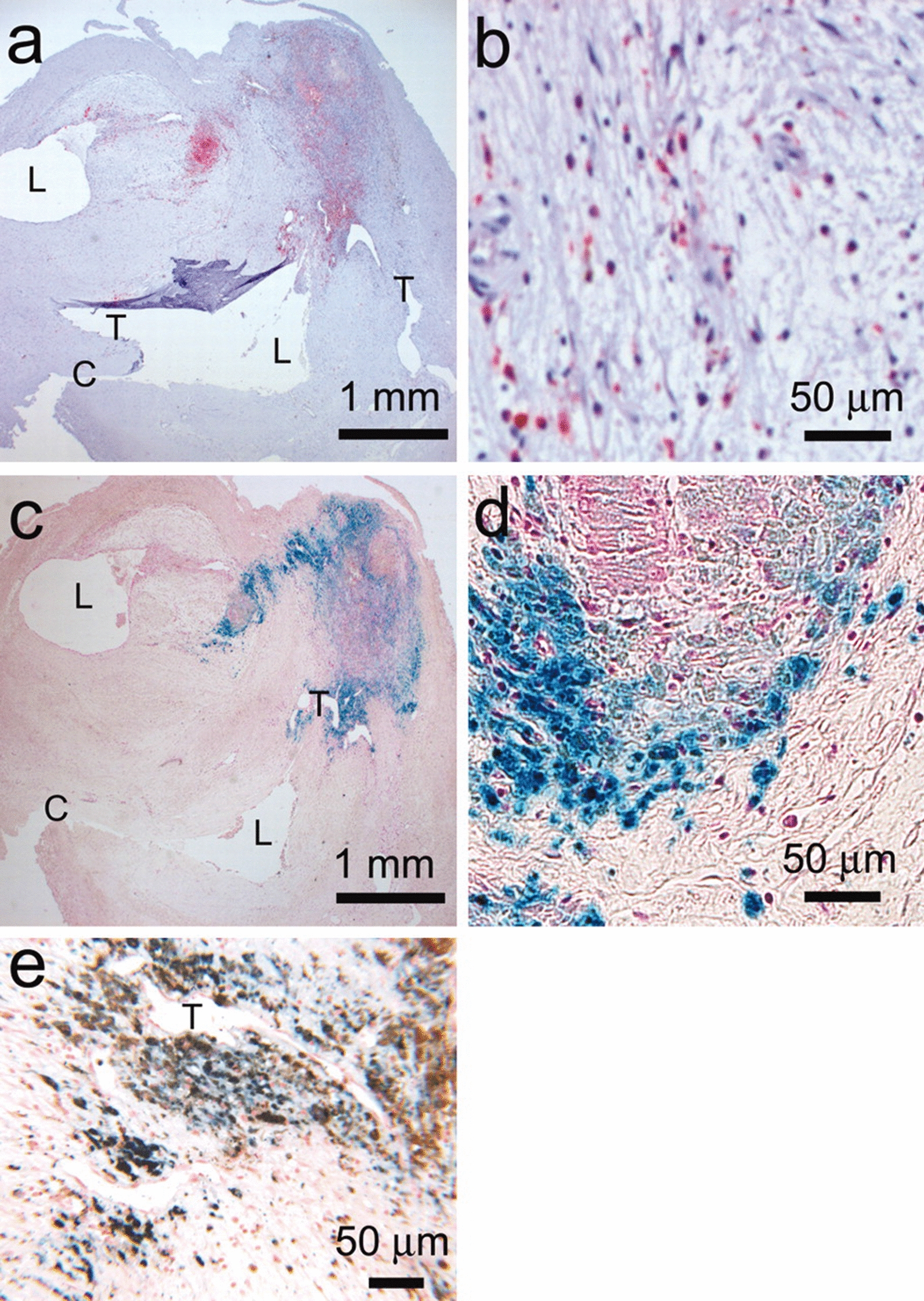
CD68 (**a** and **b**), Perl’s (**c** and **d**), and CD68/Perl’s (**e**) double staining of the endarterectomy specimen of a patient who received USPIOs. The red coloring (a and b) and brown coloring (e) are indicative of macrophages, and the blue coloring (**c**, **d**, and **e**) is indicative of the accumulation of USPIOs. The double lumen is indicated with L, the surgical cut with C, and tears with T [7]. Copyright © 2003, Wolters Kluwer Health

However, it is significant to note that NMs have also shown adverse effects on AS in recent studies. For example, the accumulation of NMs was observed in human atherosclerotic lesions [7] (Fig. 1). As exhibited in Table 1, NMs such as polymeric NPs, silica NPs (SiNPs), titanium dioxide NPs (TiO_2_ NPs) and carbon nanotubes (CNTs) have been proved to cause dyslipidemia, foam cell formation, and expansion of atherosclerotic plaques in vivo. When performing in vivo trials of NMs to induce AS, common routes of medication administration include nasal administration, nebulization, intranasal instillation, pharyngeal aspiration, intratracheal instillation, inhalation, intragastric administration, and intravenous injection (Table 1). Each of these routes has its own characteristics. For example, nasal delivery of NMs enters body circulation directly from nasal venous blood without the first-pass effect in the gastrointestinal tract and liver [8]. Delivery of NMs by pharyngeal aspiration is less efficient than intratracheal instillation, given that there is also a certain percentage of NMs that do not enter the airways after pharyngeal aspiration [9]. However, pharyngeal aspiration allows for a uniform distribution of NMs in both lungs [9]. The first-pass effects in the gastrointestinal tract and liver are unavoidable for NMs via intragastric administration. The highest bioavailability of NMs is observed via intravenous injection. Furthermore, it can be found that not only NMs that enter directly into the circulation, but also NMs via local injection induce AS. Notably, in addition to the aforementioned routes, whether other medication routes of NMs, such as dermal contact, intramuscular injection, and subcutaneous injection, can also induce AS remains to be further explored. All these studies hint that the clinical translation of NMs would be limited if we ignored the toxicological effects of NMs on AS. Although NMs have been studied to cause or exacerbate AS, this regard has not been adequately reviewed. To apply NMs more safely and effectively, we focus on the adverse effects of NMs on AS, utilizing the cellular components in atherosclerotic plaques as an entry point to elucidate the molecular mechanisms by which NMs can or may promote AS and the effects of physicochemical factors of NMs on AS-associated cells.

**Table 1:** Nanomaterials cause or aggravate atherosclerosis in vivo

Nanomaterial	Size	Animal model	Route, dose and time	Effects on atherosclerosis	References
TiO_2_ NPs	5–6 nm	CD-1 mice	Nasal administration, 1.25, 2.5, and 5 mg/kg, every day for 36 weeks	Serum TG, TC, and LDL-C ↑, HDL-C ↓; serum AGEs, ROS, Nox4, CRP, ET-1, TF, ICAM-1, VCAM-1, MCP-1, PAI-1, and t-PA ↑; fibrous cap formation; foamy cell and inflammatory cell infiltration	[172]
	12/21.6/288 nm	ApoE^−/−^ mice	Intratracheal instillation, 0.5 mg/kg, once a week for 4 weeks	Atherosclerotic lesion area ↑	[173]
	5–10 nm	ApoE^−/−^ mice	Intratracheal instillation, 10 μg, 50 μg and 100 μg/mouse/week, twice a week for 6 weeks	Serum TC ↑ and HDL-C ↓; atherosclerotic lesion area ↑; endothelial dysfunction: NO and eNOS ↓	[174]
ZnO NPs	30 nm	Wistar rats	Intratracheal instillation, 1.25, 2.5, and 5.0 mg/kg, once a week for 12 weeks	Serum TC, and LDL↑, HDL-C ↓; serum HO-1 and PECAM-1 ↑; atherosclerotic lesion area ↑; EC damage, VSMC proliferation and migration	[175]
	17 nm	Wistar rats	Intragastric administration, 10 mg/kg, every day for 12 weeks	Serum TC ↑; lipid deposits and fatty streaks in aorta; blood pressure ↑	[176]
SiNPs	59.9 nm	ApoE^−/−^ mice	Intratracheal instillation, 1.5, 3.0, and 6.0 mg/kg, once a week for 12 weeks	Serum TG, LDL-C and HDL-C ↓; atherosclerotic lesion area ↑; macrophage infiltration and ER stress in plaque	[171]
	5–35 nm	ApoE^−/−^ mice	Nebulization, 0.6 mg/mL, 5 h/day, 5 days/week for 16 weeks	Necrotic core area in the lesions ↑; M1-like macrophages (iNOS + F4/80 +) ↑	[177]
	~ 15 nm	ApoE − / − mice	Intranasal instillation, 10 mg/kg, every other day for 3 months	Atherosclerotic lesion area ↑; plaque lipidation and lipid storage of aortic wall; inflammatory cells infiltration	[178]
In_2_O_3_ NPs	35.8 ± 1.1 nm	Ldlr^−/−^ mice	Pharyngeal aspiration, 30 and 120 mg/mouse, 10 weeks after a single aspiration	Serum TC, and LDL↑; the expression of IL-6 and MCP-1 ↑; atherosclerotic lesion area ↑; the accumulation of macrophages ↑	[179]
Ni(OH)_2_ NPs	5 nm	ApoE^−/−^ mice	Inhalation, 100 μg Ni/m^3^, 5 h/day, 5 days/week for 1 week or 20 weeks	The expression of Ccl-2, Vcam-1 and Cd68 ↑; atherosclerotic lesion area ↑	[180]
Se NPs	23.1/40.4/86.8 nm	ApoE^−/−^ mice	Intragastric administration, 50 μg/kg, every day for 24 weeks	Serum TG, TC, and LDL-C ↑, HDL-C ↓; serum MDA ↑ and SOD, GPx ↓; atherosclerotic lesion area ↑; foam cells, lipids, and proliferated and randomly arranged VSMCs;	[181]
Polymeric NPs	92.69 ± 3.1 nm	ApoE^−/−^ mice	Intravenous injection, 10 mg/kg, once every 2 days for 4 weeks and 12 weeks	extensive plaque formation and severe stenosis; the expression of TNF-α and IL-6 ↑;	[82]
SWCNTs	2 nm	ApoE^−/−^ mice	Pharyngeal aspiration, 10 μg/mouse, once every other week for 10 weeks	Atherosclerotic lesion area ↑	[182]
	1–4 nm	ApoE^−/−^ mice	Pharyngeal aspiration, 20 µg/mouse Once every other week for 8 weeks	Atherosclerotic lesion area ↑	[183]
MWCNTs	3.5 nm	ApoE^−/−^ mice	Pharyngeal aspiration, 40 μg/mouse, once every other week for 10 weeks	Atherosclerotic lesion area ↑	[182]
	30 nm	ApoE^−/−^ mice	Intratracheal instillation, 25.6 μg/mouse, once a week for 5 weeks	Atherosclerotic lesion area ↑	[184]
	30 nm	Sprague Dawley rat	Intravenous injection, 50, 100 and 200 μg/kg, twice per week for 12 weeks	Atherosclerotic lesion area ↑; lipid deposition in the aortic intima; aortic calcification; endothelial cell damage and autophagy inhibition	[185]

TG, triglycerides; TC, total cholesterol; LDL-C, low-density lipoprotein cholesterol; HDL-C, high-density lipoprotein cholesterol; AGEs, advanced glycation end products; Nox4, NAD(P)Hoxidases4; CRP, c-reaction protein; ET-1, endothelin-1; TF, tissue factor; ICAM-1, intercellular adhesion molecule-1; VCAM-1, vascular cell adhesion molecule-1; PAI-1, plasminogen activator inhibitor-1; t-PA, tissue plasminogen activator; ZnO, zinc oxide; HO-1, heme oxygenase-1; PECAM-1, platelet endothelial cell adhesion molecule-1; In_2_O_3_, indium oxide; Ni(OH)_2_, nickel hydroxide; Ccl-2, chemokine (C–C motif) ligand 2; Se, selenium; MDA, malonaldehyde; SOD, superoxide dismutase; GPx, glutathione peroxidases; SWCNTs, single-walled carbon nanotubes; MWCNTs, multi-walled carbon nanotubes

## Vascular cell pathways

### Endothelial cell pathway

AS is an inflammatory disease with disturbed endothelial homeostasis. Endothelial cell (EC) dysfunction is the initiating event in AS [10]. NMs that enter the circulation lead to endothelial dysfunction through endothelial leakage and proinflammatory activation.

#### Endothelial leakage

The integrity of endothelial layer is regulated by different types of cell–cell junctions. Once the junctional complex is disrupted by NMs, the gap created between two adjacent cells leads to a leakage, which is known as NM-induced endothelial leakage [11]. Endothelial leakage, which is a precursor of atherosclerotic lesions, further leads to the extravasation of macromolecules such as lipoproteins and leukocytes into the interstitial tissue, causing subendothelial lipid accumulation and inflammatory responses [10].

**Fig. 2:**
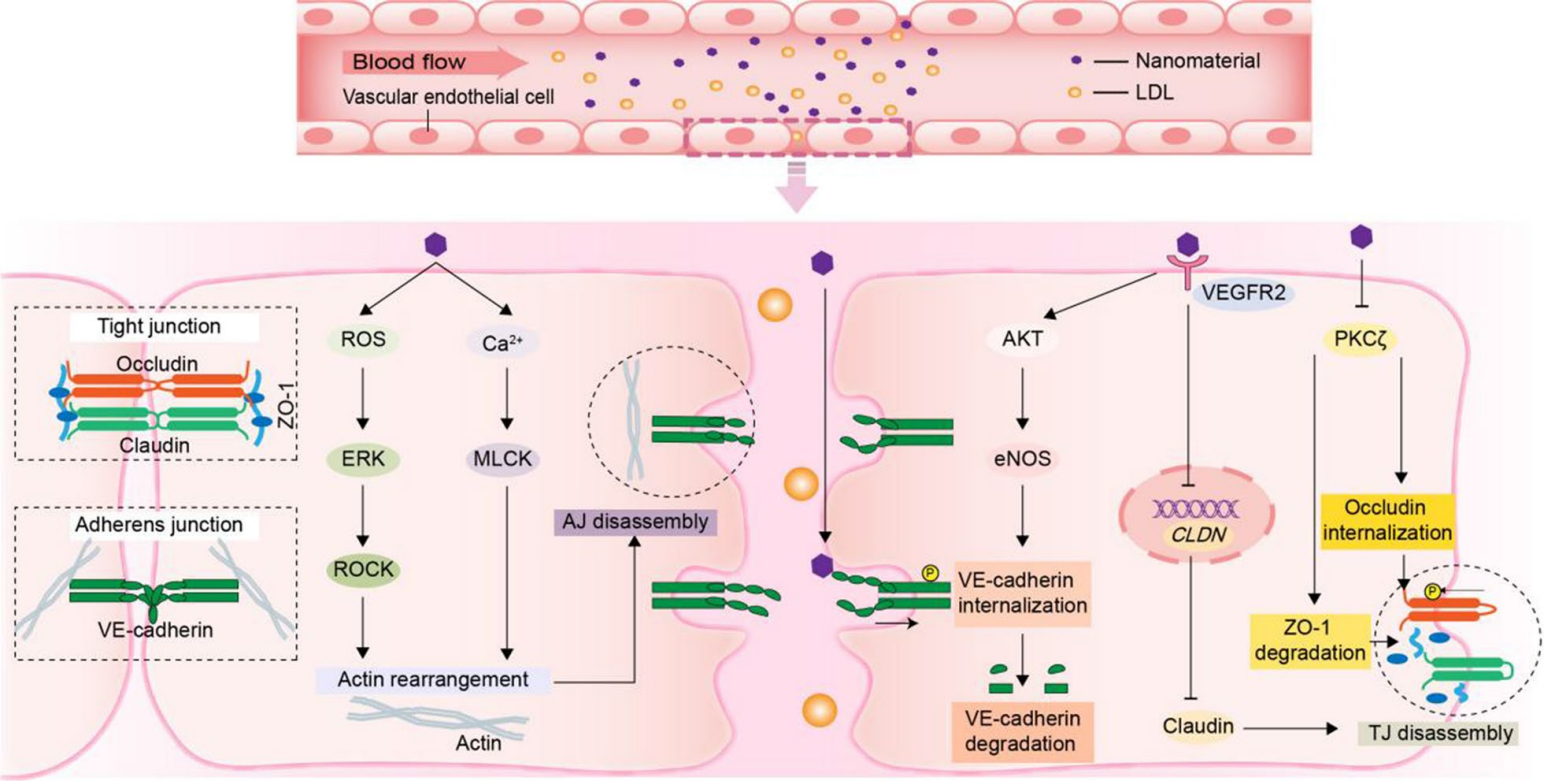
Schematic of the main mechanisms of NM-induced endothelial leakage. NMs cause endothelial leakage by disrupting VE-cadherin, claudins and occludin and inducing actin rearrangements, leading to subsequent exudation of LDL and leukocytes

As shown in Fig. 2, NMs cause endothelial leakage by disrupting tight junctions (TJs) and adherens junctions (AJs). The membrane proteins claudin and occludin and the scaffolding protein zonula occludens-1 (ZO-1) are the main proteins constituting TJs [12]. NMs significantly reduce the expression of claudin-5 by activating vascular endothelial growth factor receptor 2 (VEGFR2), which in turn perturbs the continuous distribution of claudin-5 in endothelial junctions, leading to endothelial leakage [13]. Additionally, NMs promote the internalization of occludin and the degradation of ZO-1 by inhibiting protein kinase C (PKC)ζ-mediated threonine phosphorylation, which hinders the interaction between occludin and ZO-1, leading to endothelial leakage [14]. Interestingly, membrane cholesterol also plays an important role in the structure and function of TJs. A reduction in cholesterol mediates the internalization of claudins and eliminates the formation of TJs [15]. NMs have been shown to regulate the level of membrane cholesterol [16]. However, whether NMs can cause endothelial leakage by decreasing the level of membrane cholesterol has not yet been reported.

In addition, vascular endothelial (VE)-cadherin is a major transmembrane component of AJs that binds as a cis-dimer at the cell surface and forms homophilic interactions between neighboring cells via extracellular structural domains. Studies have examined that NMs directly migrate to the extracellular structural domain of VE-cadherin, disrupting the homophilic interaction of VE-cadherin [11]. Moreover, NMs activate the protein kinase B (Akt)/endothelial-type nitric oxide synthase (eNOS) signaling pathway by binding to VEGFR [17], causing the phosphorylation, internalization and degradation of VE-cadherin [18]. Both of these mechanisms lead to endothelial leakage. Moreover, cytoskeletal rearrangement is also one of the mechanisms by which NMs cause endothelial leakage. For example, NMs induce actin cytoskeletal rearrangement and subsequent AJ depolymerization, leading to endothelial leakage through the reactive oxygen species (ROS)/extracellular signal-regulated kinase (ERK)/Rho-associated kinase (ROCK) signaling pathway [11, 18] and Ca^2+^/myosin light chain kinase (MLCK) signaling pathway [19]. Notably, NMs may also mediate cytoskeletal rearrangement through the junction matrix instead of intracellular signaling, triggering endothelial leakage. NMs have been reported to induce disassembly of the junction matrix [20], which is strongly connected to the cytoskeleton. However, this possibility still needs further experimental confirmation in ECs.

#### Proinflammatory activation

Aside from endothelial leakage, NMs induce an inflammatory response in ECs, i.e., proinflammatory activation. This inflammation is characterized by EC expression and secretion of various inflammatory mediators, such as tumor necrosis factor-α (TNF-α), interleukin (IL)-6, IL-8, monocyte chemotactic protein-1 (MCP-1), intercellular adhesion molecule-1 (ICAM-1), and vascular cell adhesion molecule 1 (VCAM-1). These substances attract macrophages and neutrophils, which attach to activated endothelial cells and penetrate the arterial wall, aggravating inflammation and promoting the progression of AS [10].

The present research has explored that NMs lead to proinflammatory activation in ECs through oxidative stress. Specifically, NMs induce oxidative stress in ECs through three pathways. First, NMs upregulate inducible nitric oxide synthase (iNOS) and downregulate eNOS expression in ECs, resulting in high levels of NO production [21]. Second, NMs increase the production of oxidative stress products in ECs such as superoxide anion radicals (-O^2−^), hydrogen peroxide (H_2_O_2_) and hydroxyl radicals (OH-) through the peroxisome proliferator-activated receptor-γ coactivator (PGC)-1α/nuclear respiratory factor 1 (NRF1)/mitochondrial transcription factor A (TFAM) signaling pathway, which is associated with mitochondrial dysfunction [22]. Finally, NMs activate the mitogen-activated protein kinase (MAPK)/nuclear factor erythroid 2-related factor 2 (Nrf2) signaling pathway, leading to oxidative stress in EC [21].

Furthermore, NMs induce proinflammatory activation in ECs through NOD-like receptor thermal protein domain associated protein 3 (NLRP3) inflammasomes and autophagy. For example, NMs activate the high-mobility group protein B1 (HMGB1) /toll-like receptor 4 (TLR4)/myeloid differentiation primary response 88 (MyD88) signaling pathway by triggering NLRP3 inflammasomes, leading to proinflammatory activation of ECs [23]. NMs promote C-reactive protein (CRP) and IL-1β production by enhancing autophagic activity and blocking autophagic flux, leading to proinflammatory activation of ECs [24]. Intriguingly, there may also be an association between these two pathways, as NLRP3 inflammasomes can enhance autophagy [25]. Therefore, this interaction between NLRP3 inflammasomes and autophagy could also be a potential mechanism by which NMs mediate proinflammatory activation of ECs.

### Smooth muscle cell pathway

After impairing endothelial integrity, NMs cross the damaged vascular endothelium and reach the subendothelial space, where they encounter vascular smooth muscle cells (VSMCs), leading to VSMC phenotypic switching, proliferation and migration, which are important pathological processes in AS and affect plaque formation, development and stability.

#### Phenotypic switching

**Fig. 3:**
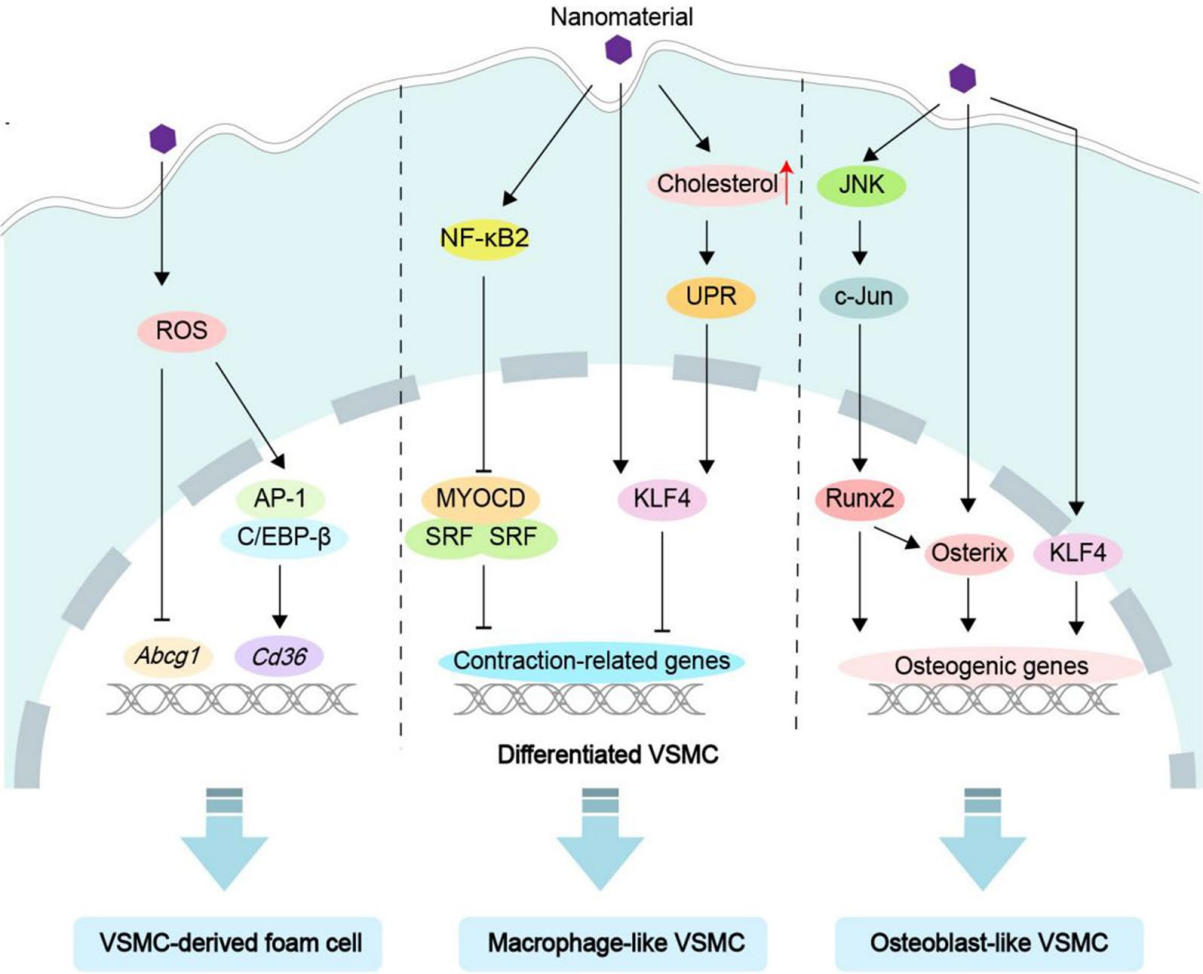
Schematic of the main mechanisms of NM-induced phenotype switching. NMs promote VSMCs from the contractile type to foam cells, macrophage-like VSMCs, and osteoblast-like VSMCs

As observed in Fig. 3, NMs promote VSMCs to undergo phenotypic switching from contractile VSMCs to foam cells, macrophage-like VSMCs and osteoblast-like VSMCs.

##### Foam cells

More than 50% of foam cells in atherosclerotic plaques are derived from VSMCs [26]. Current studies have suggested that NMs lead to VSMC-derived foam cell formation via ROS [27]. On the one hand, NM-induced ROS can increase the expression of scavenger receptor A (SR-A) by activating transcription factor activating protein-1 (AP-1) or CCAAT/enhancer binding protein beta (C/EBPβ), thus promoting LDL uptake by VSMCs [28]. On the other hand, NM-induced ROS can reduce cholesterol efflux by inhibiting transcription of the cholesterol efflux transporter ATP binding cassette subfamily G member 1 (ABCG1) [29]. Both of these processes are essential in lipid accumulation and the formation of VSMC-derived foam cells. VSMC-derived foam cells are subsequently deposited in the subintimal space of arteries, contributing to lipid streak lesions and plaque formation. Moreover, NMs promote VSMC-derived foam cell formation by lipogenesis through sterol regulatory element-binding proteins (SREBPs) [27]. However, the molecular mechanism of SREBPs activation by NMs is still unclear. It may be related to endoplasmic reticulum stress because SREBPs are located on the endoplasmic reticulum membrane and can be activated by endoplasmic reticulum stress [30], and endoplasmic reticulum stress is one of the common cellular effects of NMs [31].

##### Macrophage-like VSMCs

Macrophage-like VSMCs have decreased abilities to remove lipids, dead cells, and necrotic debris, exacerbating inflammation and leading to AS [32]. Studies have found that NMs induce VSMCs to transform to a macrophage-like phenotype through transcription factors. For example, NMs promote macrophage-like VSMC formation by inhibiting the transcription factor myocardin (MYOCD) through the atypical NF-κB family member NFκB2 [33, 34]. Besides, NMs induce VSMC conversion to a macrophage-like phenotype via the transcription factor Kruppel-like factor 4 (KLF4). This process can be associated with cholesterol loading. NM-induced cholesterol loading [33] may increase KLF4 expression by activating endoplasmic reticulum unfolded protein responses [35]. Finally, serum response factor (SRF) is also a crucial transcription factor that regulates macrophage-like VSMCs [36]. However, there is a lack of evidence of the effect of NMs on SRF. Whether NMs can promote the formation of macrophage-like VSMCs and the progression of AS by inhibiting SRF requires further examination.

##### Osteoblast-like VSMCs

Osteoblast-like VSMCs are involved in the calcification of atherosclerotic plaques [37]. Researchers found that NMs such as hydroxyapatite NPs induced VSMC transformation into an osteoblast-like phenotype. For example, NMs activate the osteogenic transcription factor runt-related factor 2 (Runx2) via the c-Jun N-terminal kinase (JNK)/c-JUN signaling pathway, which induces VSMCs to express the osteogenic markers osteopontin (OPN) and alkaline phosphatase (ALP) [38]. Additionally, NMs promote the formation of osteoblast-like VSMCs via the Runx2 downstream transcription factor Osterix [39]. Interestingly, NMs may promote the transformation of VSMCs to an osteoblast-like phenotype by the transcription factor KLF4 [33], which acts independently of Runx2 and upregulates the transcriptional activity of the osteogenic gene OPN [40]. Therefore, exploring whether the promoter regions of other osteogenic-related transcription factors, such as bone morphogenetic proteins, Msx2 and Osterix, contain binding sites for KLF4 could be the next step for research. In addition, transforming growth factor (TGF)-β is vital to osteoblast-like VSMC formation [41]. NMs have been reported to activate the TGF-β signaling pathway [42]. Therefore, it can be inferred that NMs may induce the formation of osteoblast-like VSMCs through the TGF-β signaling pathway, but there is still a lack of relevant research.

#### Proliferation and migration

In addition to phenotypic switching, NMs promote VSMC proliferation and migration. VSMC proliferation causes diffuse intimal thickening in vessels, leading to plaque growth and vascular occlusion [43]. The extracellular matrix (ECM), which surrounds VSMCs, activates integrins, leading to VSMC proliferation [44]. On the one hand, NMs can contain the same fibrous structure as the ECM and exhibit similar topography to the ECM, such as pores, grooves and ridges, to mimic ECM binding to integrins, promoting VSMC proliferation [45]. On the other hand, NMs can directly activate or increase the expression of integrin proteins [46, 47] and later activate the downstream focal adhesion kinase (FAK)/steroid receptor coactivator (Src) and Ras/PI3K/MAPK signaling pathways, ultimately leading to VSMC proliferation [48, 49]. Moreover, NMs facilitate VSMC proliferation via calcium (Ca^2+^) entry, which acts as a second messenger to maintain the cell cycle by activating intracellular proliferation-related kinases and phosphatases [50]. This Ca^2+^ influx can be the result of NM-induced mechanical stress by impinging on the cell membrane, which activates transient receptor potential vanilloid (TRPV) cation channels [51], or NM-mediated stimulation of voltage-gated calcium channels through membrane potential modification [50] or ion shedding mechanisms [52]. Finally, growth factors may also be involved in NM-induced VSMC proliferation. NMs can stimulate ECs to secrete fibroblast growth factor (FGF) and macrophages to secrete platelet-derived growth factor (PDGF) and TGF-β [53, 54]. These growth factors later regulate cyclins such as Cyclin D1 and CDK4 to promote VSMC proliferation [55–57]. However, this paracrine mechanism still needs to be further validated in NM-exposed coculture systems or in vivo.

The migration of VSMCs from the intermediate to the intimal layer is also a critical event in AS [43]. NMs adsorbing ECM components, such as fibronectin (FN) and OPN [58, 59] can activate the TLR4/Akt1/mammalian target of rapamycin (mTOR) signaling pathway and integrin/MAPK/cAMP-response element binding protein (CREB) signaling pathway, leading to VSMC migration [60, 61]. Additionally, NMs stimulate the production of matrix metalloproteinases (MMPs) [62], which remove the basement membrane around VSMCs and facilitate VSMC contact with the interstitial matrix [43]. Moreover, NMs promote VSMC migration through microtubule and actin rearrangement. For example, NMs such as multilayered 3D nanostructures induce polarization of intracellular microtubule organizing centers by mimicking nanoscale topographies such as grooves, ridges, and pores in the extracellular environment [63], which leads to the alignment of microtubule networks with cell migration axes, the protrusion of plasma membranes, and the assembly/disassembly of adhesion foci, thus promoting VSMC migration [64]. NMs such as zeolitic imidazolate framework-8 NPs lead to actin polymerization by releasing Zn^2+^ to bind to G-actin, inducing VSMC lamellar pseudopod formation and promoting cell migration [65]. Significantly, intermediate filaments, which are components of the cytoskeleton, also induce VSMC migration by regulating focal adhesion dynamics, cell contraction and nuclear stiffness [56]. NMs have been explored to regulate intermediate filament rearrangement [66]. However, whether this regulatory mechanism can promote VSMC migration and subsequently lead to AS remains obscure and requires further confirmation.

## Immune cell pathways

Aside from vascular cells, various immune cells are recruited and mobilized to atherosclerotic plaques. The mechanisms by which NMs promote AS through the innate immune cell pathway (neutrophils, macrophages, and mast cells) and the adaptive immune cell pathway (dendritic cells and T lymphocytes) will be discussed below.

### Innate immune cell pathways

#### Neutrophil pathway

There is a positive correlation between neutrophil counts in peripheral blood and atherosclerotic lesion size [67]. Neutrophils cause or exacerbate AS through respiratory bursts, neutrophil extracellular trap (NET) formation and degranulation, which leads to the release of granule proteins such as myeloperoxidase (MPO), MMPs and neutrophil elastase (NE) and oxidants such as ROS and NADPH oxidase [68]. NMs are also closely related to neutrophils and thus have proatherogenic potential.

NMs may promote AS by respiratory burst and the formation of NETs. Respiratory burst is a process in which neutrophils rapidly consume oxygen and release -O^2−^, H_2_O_2_ and OH-, leading to ROS production [69]. NMs cause a respiratory burst by damaging the membrane integrity of neutrophils and triggering the influx of Ca^2+^, which successively activates the PKC/p38 MAPK/JNK signaling pathway, leading to the reorganization and activation of NADPH oxidase components [70]. The products of the respiratory burst subsequently oxidize LDL, which induces the formation of foam cells in plaques. In addition, NETs are reticular fibrous structures with a backbone of deconcentrated nuclear chromatin (mainly DNA and positively charged histones) decorated with neutrophil-derived nuclear, cytoplasmic and granule protein components [68]. Studies have demonstrated that NMs stimulate NET formation by generating ROS through NADPH oxidase-dependent and NADPH oxidase-independent pathways. For one thing, NMs can coordinate with LPS to activate NADPH oxidase and generate ROS through the SRA/p38 MAPK/ERK signaling pathway [71]. For another, NMs can induce ROS production independently of NADPH oxidase directly through lysosomal membrane leakage [72] and through mitochondrial membrane proteins, such as reactive oxygen species modulator 1 (ROMO1) [71]. ROS subsequently activate NE and peptidyl arginine deiminase 4 (PAD4), which causes histone citrullination and chromatin deconcentration, mediating the formation of NETs [68]. In addition to ROS, NMs can induce autophagy through the PI3K/mTOR signaling pathway, leading to the formation of NETs [73]. NETs later regulate intraplaque protein hydrolysis, fibrous cap thinning, platelet activation and aggregation, thus promoting plaque instability and thrombus formation [68]. Notably, the varieties of NETs are diverse, with suicidal, vital, and mitochondrial types. Therefore, we can infer that NMs may induce the formation of different categories of NETs, but little research has been done in this aspect. Further experiments are needed to determine whether there is sufficient evidence to prove this hypothesis.

NMs may also promote AS by activating neutrophil degranulation. A study has indicated that NMs cause plasma membrane depolarization and intracellular sodium and calcium overload by releasing metal cations, which stimulate the release of mitochondrial contents and activate the phosphorylation of p38 MAPK, leading to neutrophil degranulation and the production of MPO, MMP, and NE [74]. These granule proteins have proinflammatory properties and support LDL oxidation, subsequently promoting AS. In particular, MPO released from neutrophils can also degrade NMs. This degradation may have a "secondary effect" on AS, but it depends on the degradation degree of NMs. For example, the incomplete degradation products of SWCNTs, such as oxidized aromatic hydrocarbons, cause inflammation and genotoxicity, whereas the complete degradation products, CO2 and H2O, do not cause cytotoxicity [75]. Therefore, further evaluation of the degradation products of NMs on AS is imperative in the future. In addition, NMs lead to neutrophil degranulation by inducing actin polymerization [76]. Interestingly, myosin is another decisive factor that regulates cell degranulation [77]. NMs can stimulate myosin through myosin light chain kinase (MLCK) [19]. Therefore, it can be inferred that NMs may induce neutrophil degranulation via myosin, but this mechanism needs further experimental confirmation.

#### Macrophage pathway

Macrophages, which are major sources of inflammatory factors in atherosclerotic plaques and the main immune cells in the innate immune response, play a fundamental role in the progression of atherosclerosis. NMs can promote AS by inducing the formation of macrophage-derived foam cells and polarization.

##### Foam cell formation

Macrophage-derived foam cells are significant constituents of atherosclerotic lesions and are involved in the formation and expansion of lipid cores, leading to plaque instability and rupture [78]. Macrophages exposed to NMs can be converted into lipid-rich foam cells. This process is mainly associated with LDL uptake and cholesterol efflux through cholesteryl ester hydrolysis and reverse cholesterol transport.

**Fig. 4:**
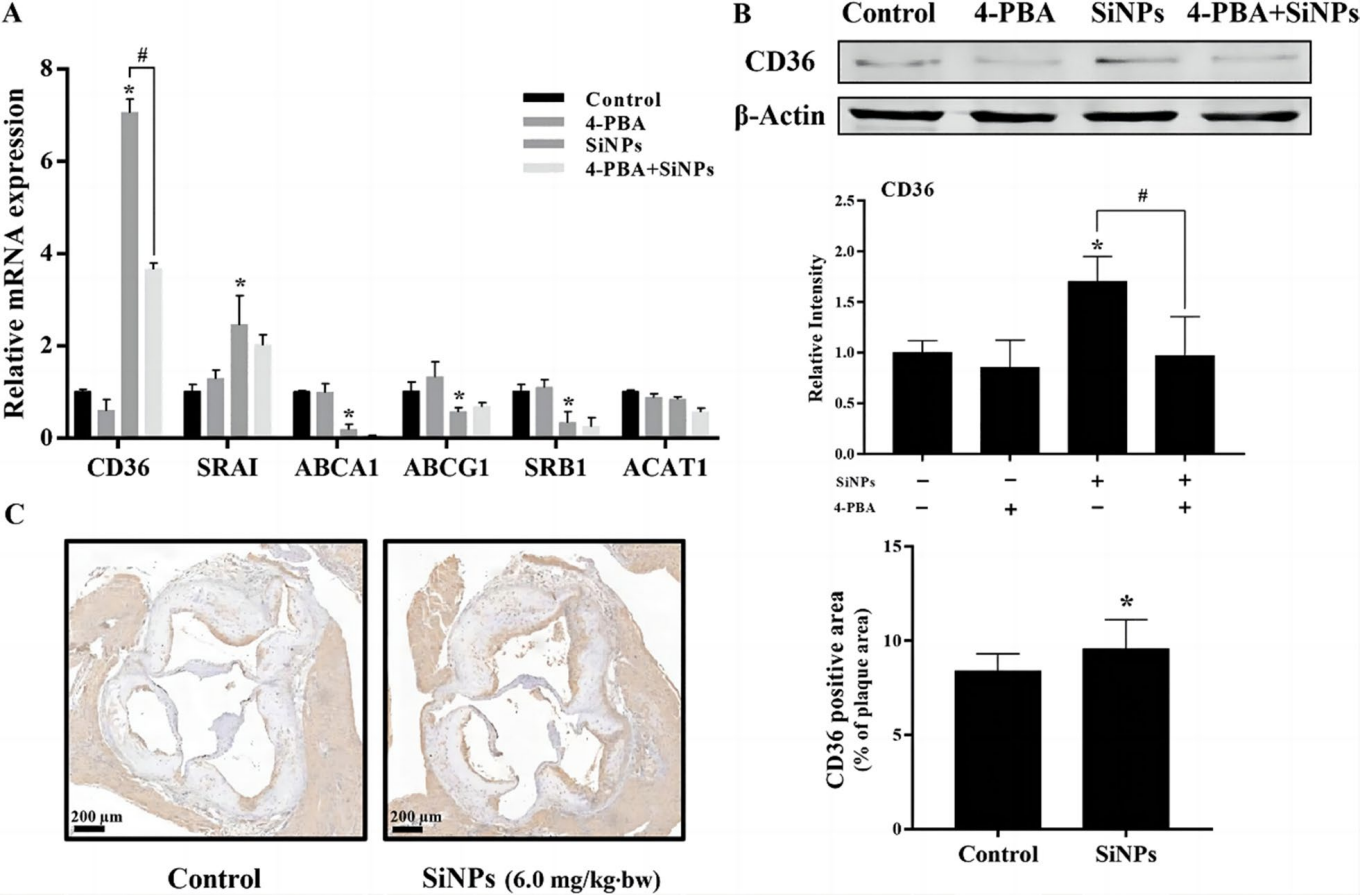
ER stress-mediated the upregulated CD36 expression attributed to the lipid accumulation induced by SiNPs in RAW264.7 cells. **A** The relative mRNA expression of factors involved in cholesterol influx/efflux. **b** CD36 protein expression in RAW264.7 cells. Consistent with the mRNA level, the upregulated CD36 expression induced by SiNPs was greatly alleviated by 4-PBA pretreatment (an ER stress inhibitor). **c** CD36 expression in the plaques of SiNP-exposed aortic roots was upregulated [171]. No Copyright

Studies have shown that NMs mediate LDL uptake and induce macrophage-derived foam cell formation through three pathways of endocytosis. First, NMs can enhance the fluid-phase pinocytosis of macrophages by activating phosphatidylinositol 3-kinase (PI3K), which leads to LDL uptake [79]. Second, NMs can upregulate lectin-like ox-LDL receptor 1 (LOX-1) expression through NF-B and increase CD36 expression through endoplasmic reticulum stress [80, 81] (Fig. 4). These scavenger receptors LOX-1 and CD36 take up LDL and induce foam cell formation. Third, NMs such as PLGA NPs can boost the phagocytosis of LDL by macrophages [82]. This mechanism may be related to lipid synthesis since NMs can promote lipid synthesis [27] and lipids can reinforce phagocytosis by maintaining the connection between the cytoskeleton and the plasma membrane [83].

In addition, NMs induce macrophage-derived foam cell formation by inhibiting cholesteryl ester hydrolysis. For example, NMs such as SiO_2_ NPs suppress cholesteryl ester hydrolysis by reducing the expression of neutral lipases such as adipose triglyceride lipase (ATGL) and hormone-sensitive lipase (HSL), leading to cholesteryl ester accumulation and foam cell formation in macrophages [84]. Moreover, cholesteryl ester hydrolysis is dependent on lysosomal acid lipase (LAL) in lysosomes [85]. NMs can be delivered to lysosomes after being endocytosed by macrophages, and this process impairs lysosomal alkalization and lysosomal membrane permeability [86]. Through this mechanism, NMs can decrease LAL activity and expression in the lysosome, which subsequently inhibits cholesteryl ester hydrolysis and leads to the formation of foam cells.

Finally, reverse cholesterol transport is also involved in the NM-induced formation of macrophage-derived foam cells. Reverse cholesterol transport is the process that transports free cholesterol out of the cell and is primarily regulated by ABC transporters, such as ABCG1 and ATP binding cassette subfamily A member 1 (ABCA1). Studies have indicated that NMs inhibit the expression of ABCG1 and ABCA1 through two mechanisms. On the one hand, NMs can reduce the expression of ABCA1 and ABCG1 through endoplasmic reticulum stress [81]. On the other hand, NMs such as graphene and graphene oxide can interrupt the plasma membrane through their sharp edges, changing the conformation of the transmembrane ABC transporter and downregulating its activity [87]. These outcomes later suppress reverse cholesterol transport, promoting the formation of macrophage-derived foam cells. In addition, the transcription factor liver X receptor (LXR) and the ubiquitin–proteasome degradation pathway are also significant in regulating ABCG1 and ABCA1 expression [88]. However, there is a lack of studies on whether NMs can regulate LXR expression and ABC transporter ubiquitination. Furthermore, NMs can inhibit reverse cholesterol transport and promote macrophage-derived foam cell formation by inducing acute phase response. For example, lung exposure to NMs can increase the blood level of acute phase protein serum amyloid A (SAA), which correlates with the neutrophil influx, retention surface area, and dose of NMs [89, 90]. Elevated SAA is subsequently incorporated into high-density lipoprotein (HDL) and replaces apolipoprotein A-1, thereby blocking HDL-mediated reverse cholesterol transport and accelerating the formation of foam cells and atherosclerotic plaques [91, 92].

##### Polarization

Macrophages regulate the inflammatory state and the stability of atherosclerotic plaques through distinct polarization types. NMs may induce plaque formation and instability by stimulating the formation of proinflammatory M1 macrophages, leading to the production of proinflammatory factors such as TNF-α, IL-6, interferon (IFN)-β and C–C motif chemokine receptor 7 (CCR7) [93].

Studies have demonstrated that NMs induce the polarization from M0 macrophages to M1 macrophages through multiple signaling pathways. For example, NMs activate the MyD88/NF-κB signaling pathway through TLR4, leading to enhanced transcription of the M1 macrophage markers TNF-α and IL-6 [94], or promote the internalization of TLR4 and CD14 dimerization, which activates the TIR-domain-containing adapter-inducing IFN-β (TRIF)/interferon regulatory factor (IRF) 3 signaling pathway, leading to the upregulation of M1 macrophage-associated target gene IL-1β expression [95]. Furthermore, NMs stimulate macrophage expression of M1-type markers such as CD86 and iNOS via the Janus kinase (JAK)/signal transducer and activator of transcription (STAT) 1 signaling pathway [96].

In addition, the chemical composition of NMs influences the M1 polarization of macrophages. For example, Fe_3_O_4_ NPs induce M1 macrophage formation by promoting intracellular Fe accumulation, leading to the ubiquitination of TNF receptor associated factor 6 (TRAF6), which activates the expression of the downstream transcription factor IRF5 and the target gene IL-23 [97]. Copper-containing bioceramic-coated titanium implants release Cu^2+^, which induces NF-κB phosphorylation by activating the copper transporter 1 (CTR1)/ATP7 signaling pathway, leading to M1 macrophage polarization [93]. Moreover, the topography of NMs provides physical stimulation to activate the FAK/MAPK signaling pathway, triggering a change in macrophage morphology from significantly elongated to round with more visible lamellar pseudopods, forming M1 macrophages [98].

Finally, NMs lead to M1 macrophage polarization through metabolic reprogramming, which is characterized by a significant increase in glycolytic activity and tricarboxylic acid cycle metabolites such as itaconate and succinate and decreased ATP production [99]. NMs such as porphyrin-iron metal-organic framework nanocrystals have acid-responsive degradation properties that generate hydrogen gas along with the release of loaded drugs. These hydrogens later downgrade oxidative phosphorylation by altering energy metabolism, such as reducing ATP production and impairing the function of mitochondria, which induces the formation of M1 macrophages [100]. Notably, most of the current research has focused on the alteration of metabolite categories and quantities, and there is still a lack of in-depth mechanistic research. Therefore, we propose to utilize transcriptomics and metabolomics to elucidate the mechanisms by which NMs regulate metabolites, which may be research directions in the future.

Indeed, in addition to M1 macrophages, M4 macrophages are also present in atherosclerotic plaques, promoting inflammation [101]. However, little attention has been given to the role of NMs on M4 macrophages in AS. Further exciting progress is expected in the exploration of NMs to regulate novel macrophage phenotypes.

#### Mast cell pathway

Mast cells are found in large numbers in the shoulder region of atherosclerotic plaques and degranulate, releasing multiple inflammatory cytokines and inflammatory mediators to induce plaque instability [102]. NMs may cause or exacerbate AS by inducing mast cell activation and degranulation.

NMs lead to mast cell activation and degranulation through IgE receptor and non-IgE receptor pathways. On the one hand, NMs can lead to mast cell activation by increasing the number and density of their loaded antigens, enhancing cross-linking and the aggregation of IgE-IgE receptor complexes [103], or promoting the phosphorylation of protein tyrosine kinase (PTK), which is downstream of IgE receptors [104]. On the other hand, NMs can trigger mast cell activation and degranulation through three non-IgE receptor pathways. First, NMs can trigger mast cell activation and the release of IL-6 and IL-8 by promoting IL-33 binding to the IL-1-like receptor ST2 [105]. IL-6 and IL-8 later promote AS by upregulating the expression of adhesion molecules on ECs and recruiting leukocytes [106]. Second, NMs can stimulate mast cell activation and the release of TGF-α, chymotrypsin and trypsin-like enzymes by activating scavenger receptors such as SR-A, SR-B1 and Marco [107, 108]. TGF-α upregulates the expression of adhesion molecules on ECs, and the main effect of chymotrypsin and trypsin-like enzymes is matrix degradation, these two lead to plaque instability [106]. Third, NMs can activate the complement system by inducing complement C5a and C3a to bind to C5a and C3a receptors on mast cells [109], stimulating histamine release from mast cells, which enhances vascular permeability and causes vascular leakage, leading to an increased risk of intraplaque hemorrhage [110].

Furthermore, studies have revealed that the non-IgE receptors activated by NMs induce mast cell degranulation mainly through calcium signaling. For example, NMs induce the opening of Ca^2+^ release-activated Ca^2+^ (CRAC) channels by binding to SR-B1 and activating the PI3K/phospholipase C (PLC) γ/diacylglycerol (DAG)/PKC signaling pathway, leading to the influx of extracellular Ca^2+^ and subsequent mast cell degranulation [108]. In addition, NMs activate the PLCγ/inositol triphosphate (IP3) signaling pathway through mitochondrial disruption and ROS production, mobilizing the release of endoplasmic reticulum Ca^2+^ and leading to mast cell degranulation [111]. All of these observations indicate that Ca^2+^ is closely connected to NM-induced mast cell degranulation. In fact, there are other regulatory mechanisms, such as Rho GTPase, cAMP, and neuro-immuno-endocrine networks, in mast cell degranulation. However, whether NMs can promote mast cell degranulation and induce AS through these pathways currently remains unknown and needs further exploration.

### Acquired immune cell pathways

#### Dendritic cell pathway

Dendritic cells (DCs), which are the most functional specialized antigen-presenting cells, link the innate and adaptive immune responses. DCs play an important role in AS by activating T cells through antigen presentation and cross-antigen presentation, stimulating the release of large amounts of inflammatory cytokines that trigger and exacerbate persistent immune damage in the arterial wall [112]. There is much relation between NMs and DCs, which may further lead to AS.

Studies have confirmed that NMs enhance DC antigen presentation. DCs recognize oxidation-specific epitopes (OSEs) and apolipoprotein A-I (APOA1) formed by oxidative modification of LDL as antigens and present them to T cells, thus promoting the differentiation of T-cell subpopulations and leading to AS [113]. On the one hand, NMs can target both DCs and T cells, acting as a "bridge" to shorten the gap between these two cells and facilitate DC antigen presentation to T cells [114]. On the other hand, NMs can enter the lysosome to limit the rate of antigen hydrolysis and to increase the duration of antigen presentation by alkalinizing the pH of the lysosome and combining free antigenic peptides, thus finally leading to sustained antigen presentation [115]. These results may lead to the differentiation of T-cell subpopulations, promoting the production of inflammatory cytokines that accelerate plaque formation.

In particular, NMs enhance cross-antigen presentation in DCs. Cross-antigen presentation is the process by which exogenous antigens are presented on MHCI molecules rather than MHCII molecules. For example, NMs induce swelling and rupture of the endosomes/lysosome through the proton sponge effect and by reducing lysosomal membrane stability, leading to the release of exogenous antigens into the cytoplasm and loading onto MHCI molecules [116]. This can lead to the activation of CD8^+^ cytotoxic T cells, which may later promote AS progression. Moreover, NMs such as cationic peptide micelles increase DC uptake of exogenous antigens via lipid raft-dependent endocytosis, which allows exogenous antigens to escape the lysosome and bind to MHCI molecules, promoting cross-antigen presentation [117].

#### T-lymphocyte pathway

Current studies have suggested that CD4^+^ and CD8^+^ T cells are critical drivers of AS pathogenesis [118]. NMs may contribute to or exacerbate AS by inducing the differentiation and functional alterations of T cells.

CD4^+^ T cells promote AS by differentiating into Th1, Th17 and Treg cells. Among them, Th1 cells are the most prominent Th-cell subpopulation in atherosclerotic plaques [119]. NMs such as [Gd@C_82_(OH)_22_] NPs induce the differentiation of CD4^+^ T cells into Th1 cells by enhancing DC maturation and antigen presentation [120]. Th1 cells subsequently secrete IFNγ and promote M1 macrophage formation, leading to AS and decreased plaque stability [121, 122]. Furthermore, NMs such as TiO_2_ NPs induce Th1-cell formation by activating CD4^+^ T cells directly [123]. Although this regulatory mechanism is not yet clear, we can infer that NMs may activate the surface receptors associated with T-cell differentiation, such as T-cell receptor (TCR), CD28 and programmed cell death 1 (PD-1), leading to the differentiation of CD4^+^ T cells into Th1 cells [124–126]. Intriguingly, NMs may promote AS by stimulating or inhibiting the formation of Th17 cells. For one thing, NMs such as carbon black can enhance the secretion of IL-6 and IL-1β by DCs through the inhibition of histone deacetylase (HDAC) 4 expression, the activation of the transcription factor AP-1, the induction of the MAPK/ERK signaling pathway and the adapter protein ASC-mediated assembly of inflammasomes, inducing the differentiation of CD4 + T cells to Th17 cells [127, 128]. These Th17 cells subsequently promote the migration of macrophages and neutrophils to the atherosclerotic lesion, leading to an inflammatory microenvironment [129]. For another, NMs such as iron oxide NPs can inhibit Th17 cell formation by reducing the expression of RAR-associated orphan receptor (ROR-γ), a transcription factor that is characteristic of Th17-cell development, and the cytokine IL-6 [130]. This may further lead to reduced type I collagen production by VSMCs and exacerbate atherosclerotic plaque instability [131]. Therefore, because of the duality of Th17 cells in AS, either upregulation or downregulation of Th17 cells influenced by NMs can lead to AS.

In addition, NMs may promote AS via CD8 + T cells. For example, NMs such as SiNPs and ceria NPs induce the differentiation of CD8^+^ T cells into cytotoxic T cells through DC cross-antigen presentation and the NF-κB signaling pathway [132, 133]. Cytotoxic T cells later secrete perforin, granzyme B, IFN-γ, and TNF-α, mediating the apoptosis of macrophages and leading to the formation of necrotic cores in plaques [134]. Notably, with the development of AS, CD8 + T cells also attenuate atherosclerotic lesions by lysing macrophages and alleviating inflammation [135]. Thus, NM-mediated activation of CD8 + T cells may not aggravate AS. Therefore, it can be inferred that the effect of NMs is not necessarily constant and may change accordingly with the progression of AS and CD8 + T-cell function. Researchers should pay more attention to the impacts of NMs on atherosclerotic plaques at different stages.

Notably, most of the current studies investigating the effect of NMs on T-cell differentiation focus on metallic NMs, which induce T-cell differentiation by activating cross-antigen presentation in DCs and signaling pathways. However, whether other NMs such as polymer-based and lipid-based NMs can also induce T-cell differentiation is unknown and requires further exploration. Finally, other T-cell subsets, such as T_FH_ cells, CD28^null^ T cells, and invariant natural killer T cells, have been reported to have proinflammatory and proatherosclerotic properties [118]. However, there is still a lack of studies on the effects of NMs on these T-cell subsets, which could be research directions in the future.

## Influence factors mediating the effects of NMs on AS-related cells

### Size

NMs that are too large or too small can have adverse effects on AS-associated cells. For T cells, 70–100 nm silica NPs induce cytotoxic T-cell formation [132], while 10 nm ultrasmall silica NPs activate the formation of Th1 cells [136]. Similarly, the size of NMs affects mast cell activation. A study indicated that larger NMs enhance mast cell activation by loading more IgE ligands because they have a greater surface area [103], while smaller NMs (5 nm) activate mast cells as well [137]. These results may be because the larger NMs are in contact with the cell membrane for a longer time, while the smaller NMs can pass directly through ion channels on the cell membrane [138, 139], affecting ion exchange, such as Ca^2+^ and leading to the activation of mast cells.

Furthermore, the effects of NM size on AS-related cells depend on subcellular localization. For example, oversized NMs, such as graphene oxide nanosheets (50–1300 nm), stimulate high levels of proinflammatory factor secretion by macrophages due to their greater tendency to adsorb to the cell membrane, leading to activation of the TLR4/NF-κB signaling pathway [140]. Meanwhile, NMs that are too small, such as polyvinylpyrrolidone sliver NPs (10 nm), cause respiratory bursts due to their access to neutrophils, damaging cell membranes and lysosomes [141]. In particular, NMs that are too small, such as TiO_2_ (23.5 nm), can directly migrate to the adhesion junctions of ECs, disrupting the homophilic interaction of VE- cadherin and thus leading to endothelial leakage [11].

### Shape

The shape of NMs is another vital factor that regulates NM-cell interactions. The shape of NMs can modulate the adhesion of NMs to VSMCs. For example, different shapes of hydroxyapatite NPs have different adhesion strengths to VSMCs (H-Rod < H-Needle < H-Sphere < H-Plate) [39]. These hydroxyapatite NMs with a greater ability to adhere to VSMCs are more likely to cause the calcification of atherosclerotic plaques [142]. This difference is associated with the contact patterns between NMs and cells, which are mainly divided into three types: point-to-plane, line-to-plane, and plane-to-plane [143, 144]. Specifically, different contact patterns lead to distinct cellular effects due to their difference in coverage areas as well as in the deformation energy needed during membrane wrapping. For example, in point-to-plane types, nanorods, nanopins, and nanospheres enter cells via clathrin-mediated endocytosis [145], while nanoplates, which are plane-to-plane types, attach to the cell membrane in parallel without phagocytosis. Therefore, it can be inferred that the difference in NM-cell membrane interactions could be one of the reasons why different shapes of NMs lead to different adhesion strengths. However, it is essential to note that similar studies are still lacking to confirm that the shape of other NMs regulates the adhesion of NMs to VSMCs in accordance with this pattern (Rod < Needle < Sphere < Plate). In addition, Ag nanowires activate more membrane receptors than spherical Ag NPs due to their high aspect ratio, leading to mast cell degranulation [146].

Notably, after translocation to the subcellular compartment, different shapes of NMs can enter the lysosome in different amounts. This result may further lead to different degrees of lysosomal damage, which could alter cellular effects such as lipid autophagy in macrophages and antigen presentation in DCs with lysosomes as the main mechanism, ultimately having different degrees of effects on AS. It has been shown that the number of nanospheres and nanoplates entering lysosomes is higher than that of nanorods and nanopins [39]. This difference could be related to the fact that nanospheres have the largest uptake rate [147] and that nanoplates have a higher level of membrane diffusion [47].

### Surface charge

Charge (cationic, neutral, anionic or amphoteric) has a significant effect on the electrostatic interactions between NMs and cell membranes. Given that cell membranes are generally negatively charged, cationic NMs have a stronger affinity for cell membranes than anionic/neutral NMs and therefore pass through them more easily. Although cationic NMs have the advantages of lysosomal escape and forming stable complexes with negatively charged DNA and can be used for gene delivery to treat AS [3], cationic NMs could also have adverse effects on AS-associated cells. For example, cationic NMs can strongly attach to cell membranes, leading to membrane rupture and lysis, thereby inducing neutrophil degranulation [148]. Moreover, the accumulation of cationic NMs in nuclear endosomes/lysosomes can lead to an influx of protons and chloride ions (proton sponge effect), causing vesicle swelling and rupture, which promotes DC cross-antigen presentation and subsequent activation of cytotoxic T cells [116]. These cells may later promote AS by producing large amounts of proinflammatory factors. Therefore, the potential pro-atherosclerotic properties of cationic NMs should be fully considered when applying cationic NMs to biomedicine.

### Surface modifications

The regulation of AS-associated cells by NMs is also associated with surface modifications. For example, surface modifications of carbohydrates and peptides facilitate the targeting of NMs to macrophages and induce their polarization toward the M1 type because carbohydrates selectively bind glycoproteins or glycobinding proteins (e.g., lecithin) in cell membranes [149], while peptides such as Arg-Gly-Asp (RGD) sequences specifically bind to integrins in cell membranes [150]. In addition, positively charged polydiallyl dimethylammonium chloride has a high attractive force for the ${PO3}_{4}^{3-}$ groups in the lipid bilayer, which promotes the binding of NMs to cells [151]. This strong binding further leads to the depolymerization of F-actin, which induces the phenotypic switching of VSMCs [152]. Moreover, polyethylene glycol (PEG) chains can adsorb to/partially insert into the membrane surface through their surface/edge (ring and end) structures, activating integrins and their downstream signaling pathways, such as FAK/RAS/ERK and PI3K/PKC/NF-κB, thus inducing inflammatory responses in macrophages [47]. Interestingly, in addition to promoting NM-cell interactions, PEG may also have a negative effect on AS by hindering NM-cell interactions. A study has indicated that PEG inserts into the surface of NMs and produces steric hindrance, preventing phagocyte-mediated recognition and degradation of NMs [153]. This can lead to the excessive accumulation of NMs, which may amplify the adverse effects of NMs on AS-associated cells.

Furthermore, the functional groups are another determining factor that modulates the degree of opening of paracellular pathways in the vascular barrier. It has been reported that nanodiamond (ND)-NH_2_ induced a greater degree of vascular endothelial leakage than ND-COOH. This is because ND-NH_2_ has a distinct NH_2_ group on its surface that binds to intracellular amine oxidase with an amine substrate, leading to more robust ROS release [18]. Finally, coatings detached from NMs have adverse effects on AS-associated cells. For example, detached cationic surfactant ligands, such as cetyltrimethylammonium bromide (CTAB), insert into phospholipid bilayers, compressing lipids aligned with shorter ligands and reducing the thickness of cell membranes [151], which can further lead to membrane rupture in neutrophils and the release of inflammatory mediators [148].

### Protein corona

**Fig. 5:**
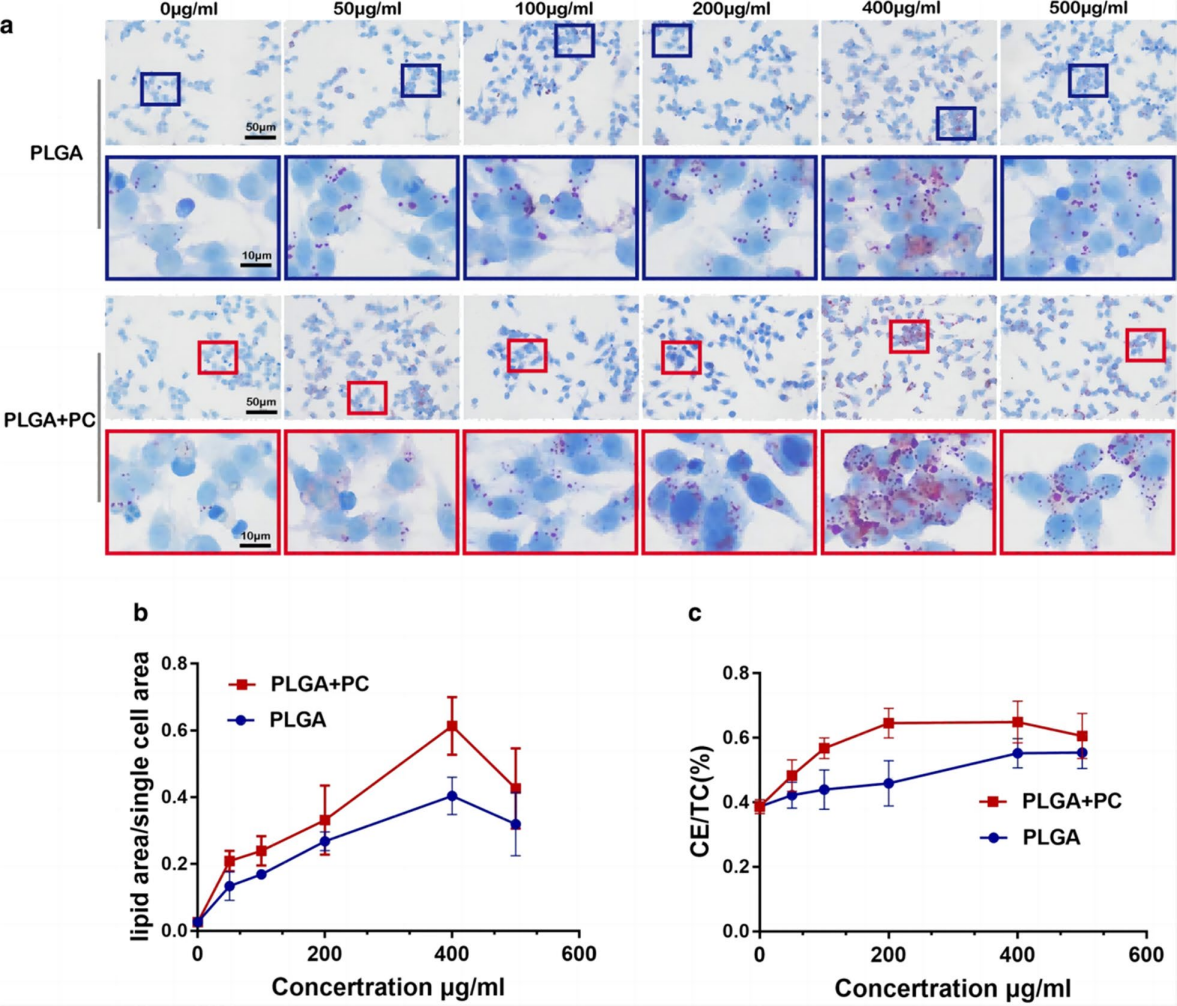
Effects of PLGA NPs and their protein coronas on the transformation of macrophages into foam cells. A, b Effects of PLGA NPs and PLGA + PC on the phagocytosis of ox-LDL by Raw 264.7 macrophages. C The CE/TC (%) of macrophages to foam cells after treatment with NPs [19]. No Copyright

NMs entering the blood or interstitial fluid can rapidly bind to proteins and form protein coronas due to their large surface area to volume ratio and high surface free energy. The protein corona has been shown to affect the cellular uptake, transport and biodistribution of NMs [154]. More importantly, the protein corona also affects NM-cell interactions, promoting the atherogenic process. For example, PLGA NPs incubated with serum to form protein coronas increased LDL uptake by macrophages to a greater extent than PLGA NPs alone, leading to foam cell formation [82] (Fig. 5). This distinction is related to the type and function of the protein corona, such as the ability of apolipoproteins to promote cellular lipid transport [155]. Moreover, a study found that the binding of CNTs to IgG could promote the specific recognition of CNTs by neutrophils and stimulate the release of MPO from neutrophils [156]. MPO subsequently promotes inflammatory responses and LDL oxidation, leading to vascular damage and foam cell formation. Intriguingly, NMs also directly affect the structure and function of the protein corona. For example, NMs bind to MPO and alter its conformation, leading to a significant increase in MPO activity [157]. In addition, enzymes from the coagulation cascade can be found in the corona surrounding NMs. NMs can activate the FXII zymogen and maintain FXIIa activity [158], which may further lead to plaque thrombosis.

### Other properties

The elasticity of NMs also contributes to regulating NM-cell interactions, promoting the development of AS. For example, rigid (3000 kPa) NMs significantly enhance the phagocytosis efficiency of macrophages compared to soft (10 kPa) NMs [159], which can further increase oxLDL uptake by macrophages and accelerate the formation of macrophage-derived foam cells. Generally, rigid NMs retain their form during cellular uptake, while soft NMs are usually deformed due to the forces associated with specific ligand-receptor interactions and membrane wrapping, leading to decreased receptor binding capacity and reduced endocytosis efficiency [160, 161]. Additionally, the density of NMs can have an impact on AS-associated cells. A research showed that NMs exacerbated endothelial leakage by increasing gravity due to increased density [11]. Increased gravity can generate an impact force to directly disrupt VE-cadherin interactions at endothelial cell junctions.

In summary, modulating the physicochemical properties of NMs to regulate the interaction between NMs and AS-associated cells is crucial. Obviously, it is not sufficient to pay attention to only one or a few of the properties of NMs, and researchers must carefully consider and incorporate a range of parameters, such as size, shape, and surface modifications, when exploring the mechanism by which NMs promote AS. Importantly, the physicochemical properties of NMs may be further altered when they come into contact with cell membranes or intracellular components [162, 163]. Whether these changes could also alter the effects of NMs on AS-associated cells still needs to be further examined.

## Strategies to avoid the proatherosclerotic properties of nanomaterials

To optimize the application of NMs in daily life and biomedicine, it is imperative to avoid the potential proatherosclerotic properties of NMs. To achieve this goal, we can consciously minimize the exposure of these NMs to the human body by specifying which NMs have toxicological effects on AS. With regard to NMs that have a wide range of biomedical applications such as drug delivery, imaging, and therapy, we can reduce the toxicological effects of NMs on AS by adjusting the physicochemical factors and surface modification of NMs.

First, the risk of NMs causing or exacerbating AS can be diminished by adjusting the physicochemical factors of NMs. For example, the size of NMs is constantly optimized, given that NMs that are either too large or too small have adverse effects on AS. In terms of surface charge, neutral and slightly negatively charged NMs should be chosen to avoid cationic NM-induced toxicity in AS-associated cells. Regarding the shape of NMs, nanoplates, nanospheres, and nanowires should be avoided. In addition, factors such as elasticity and density can also induce NMs to promote AS. However, there are few studies in this area, and more research is warranted on how to adjust these factors to minimize the toxic effects of NMs on AS.

Second, the optimized surface modification of NMs can also contribute to preventing or limiting the induction of AS by NMs. On the one hand, the interaction of NMs with AS-associated cells can be effectively curtailed by engineering NMs to precisely target the tissues and cells that need treatment as well as controlled release [164]. On the other hand, as a promising technology, cell membrane coating technology can also help circumvent the adverse effects of NMs on AS. This is because, membrane coating technology can use cell membranes such as red blood cell membranes and platelet membranes as carriers to facilitate the delivery of core NPs without being detected by immune cells and without binding to serum proteins [165]. This approach can effectively impede the mechanism of AS induction by NMs through immune cell pathways and protein corona formation. In addition, surface modification should also avoid the use of polydiallyldimethylammonium chloride, PEG, and CTAB, given that they have proven to cause toxicity in AS-associated cells.

In summary, it is crucial to effectively avoid or minimize the toxicological effects of NMs on AS to promote the sustainable development of nanomedicine. It remains an indispensable topic for the future to continuously uncover new mechanisms by which NMs promote AS and explore practical ways to circumvent their toxicological effects.

## Limitations and prospects

In conclusion, studies on the promotion of AS development by NMs have made progress. However, these studies still have some limitations.

First, the current studies mainly focus on animal experiments and lack the analysis of clinical cases. With the widespread application of NMs in sunscreen products, pharmaceuticals, cosmetics, and construction materials, large amounts of NMs are entering people's living environment, posing health risks to researchers, workers, and consumers. According to a recent study, scientists have detected ultrafine artificial particles in human thrombosis [166]. This result sheds light on the possible involvement of NMs in human AS formation. Therefore, in the future, we can review and document the history of NM exposure in AS patients during their work or life to explore the clinical relevance between NMs and AS. On the other hand, efforts should also be made to develop more advanced detection techniques to confirm the presence of NMs in the blood or the carotid intima of AS patients.

Second, most animal studies have only investigated the short-term toxicological effects of NMs. However, AS is a chronic inflammatory process, and the course of AS in humans can last for years or decades. This means that some NMs that do not promote the development of AS in the short term may also lead to AS in the future possibly because of insufficient exposure time. Therefore, it is indispensable to prolong the exposure time of NMs and to observe the long-term effects of NMs on AS. However, in this process, we also face another problem. Since there are differences between humans and rodents, we still struggle to determine exactly how many days a week of mouse exposure to nanomaterials corresponds to human exposure. Therefore, to better evaluate the long-term and chronic effects of NMs on AS in humans, we should find a more suitable animal model and establish rigorous and clear criteria for the conversion of NM exposure time from animals to humans.

In addition, in vitro experiments have some limitations. The current studies fail to cover a complete range of cell types. Aside from the cell types mentioned previously, other cell types, such as monocytes, natural killer cells (NK cells), B lymphocytes and platelets, have also played nonnegligible roles in AS [2, 167]. However, whether NMs can cause or exacerbate AS through these cells remains unclear. Additionally, no studies have investigated whether there is variability or consistency in the response between different AS-associated cells and one type of NM. It will help to select cellular models that are more sensitive to NMs and to reveal the main cellular pathways by which NMs cause or exacerbate AS.

Finally, the mechanisms by which NMs promote the progression of AS are still not fully understood. Although the effects of NMs on individual AS-associated cells have been widely discussed, studies on the promotion of AS by NMs through cell‒cell interactions are still rarely reported and need to be further explored. An organism is a system of multicellular interaction networks. Unlike individual cells, cell-cell interactions more closely reflect the true picture of AS in vivo. Furthermore, senescence is also a critical mechanism mediating the growth, inflammation and instability of AS lesions. Studies have indicated that senescence of AS-associated cells leads to degeneration and thinning of the fibrous cap and promotes plaque rupture [168, 169]. More importantly, some NMs have been reported to induce cellular senescence by perturbing telomere function [170]. Therefore, cellular senescence may be a potentially significant mechanism by which NMs cause or aggravate AS, but direct evidence is still lacking and this topic deserves more attention in future studies.

## Data Availability

Not applicable.

## Abbreviations

CVDs: Cardiovascular diseases
AS: Atherosclerosis
NMs: Nanomaterials
PLGA NPs: Poly (lactic-co-glycolic acid) nanoparticles
LDL: Low-density lipoprotein
PT: Pitavastatin
SiO_2_ NPs: Silica NPs
TiO_2_ NPs: Titanium dioxide NPs
CNTs: Carbon nanotubes
EC: Endothelial cell
TJs: Tight junctions
AJs: Adherens junctions
ZO-1: Zonula occludens-1
VEGFR2: Vascular endothelial growth factor receptor 2
PKC: Protein kinase C
VE: Vascular endothelial
Akt: Protein kinase B
eNOS: Endothelial-type nitric oxide synthase
ROS: Reactive oxygen species
ERK: Extracellular signal-regulated kinase
ROCK: Rho-associated kinase
MLCK: Myosin light chain kinase
TNF-α: Tumor necrosis factor-α
IL: Interleukin
MCP-1: Monocyte chemotactic protein-1
ICAM-1: Intercellular adhesion molecule-1
VCAM-1: Vascular cell adhesion molecule 1
iNOS: Inducible nitric oxide synthase
-O^2^^−^: Superoxide anion radicals
H_2_O_2_: Hydrogen peroxide
OH-: Hydroxyl radical
PGC: Peroxisome proliferator-activated receptor-γ coactivator
NRF1: Nuclear respiratory factor 1
TFAM: Mitochondrial transcription factor A
MAPK: Mitogen-activated protein kinase
Nrf2: Nuclear factor erythroid 2-related factor 2 l
NLRP3: NOD-like receptor thermal protein domain associated protein 3
HMGB1: High-mobility group protein B1
TLR4: Toll-like receptor 4
MyD88: Myeloid differentiation primary response 88
CRP: C-reactive protein
VSMCs: Vascular smooth muscle cells
SR-A: Scavenger receptor A
AP-1: Activating protein-1
C/EBPβ: CCAAT/enhancer binding protein beta
ABCG1: ATP binding cassette subfamily G member 1
SREBPs: Sterol regulatory element-binding proteins
MYOCD: Myocardin
KLF4: Kruppel-like factor 4
SRF: Serum response factor
Runx2: Runt-related factor 2
JNK: C-Jun N-terminal kinase
OPN: Osteopontin
ALP: Alkaline phosphatase
TGF: Transforming growth factor
ECM: Extracellular matrix
FAK: Focal adhesion kinase
Src: Steroid receptor coactivator
Ca^2+^: Calcium
TRPV: Tansient receptor potential vanilloid
FGF: Fibroblast growth factor
PDGF: Platelet-derived growth factor
FN: Fibronectin
mTOR: Mammalian target of rapamycin
CREB: CAMP-response element binding protein
MMPs: Matrix metalloproteinases
NETs: Neutrophil extracellular traps
MPO: Myeloperoxidase
NE: Neutrophil elastase
ROMO1: Reactive oxygen species modulator 1
PAD4: Peptidyl arginine deiminase 4
MLCK: Myosin light chain kinase
PI3K: Phosphatidylinositol 3-kinase
LOX-1: Lectin-like ox-LDL receptor 1
ATGL: Adipose triglyceride lipase
HSL: Hormone-sensitive lipase
LAL: Lysosomal acid lipase
ABCA1: ATP binding cassette subfamily A member 1
LXR: Liver X receptor
IFN: Interferon
CCR7: C–C motif chemokine receptor 7
TRIF: TIR-domain-containing adapter-inducing IFN-β
IRF: Interferon regulatory factor
JAK: Janus kinase
STAT: Signal transducer and activator of transcription
TRAF6: TNF receptor associated factor 6
CTR1: Copper transporter 1
PTK: Protein tyrosine kinase
CRAC: Ca^2+^ release-activated Ca^2+^
PLC: Phospholipase C
DAG: Diacylglycerol
IP3: Inositol triphosphate
DCs: Dendritic cells
OSEs: Oxidation-specific epitopes
APOA1: Apolipoprotein A-I
TCR: T-cell receptor
PD-1: Programmed cell death 1n
HDAC: Histone deacetylase
ROR-γ: RAR-associated orphan receptor
RGD: Arg-Gly-Asp
PEG: Polyethylene glycol
ND: Nanodiamond
CTAB: Cetyltrimethylammonium bromide
